# DroID: the *Drosophila *Interactions Database, a comprehensive resource for annotated gene and protein interactions

**DOI:** 10.1186/1471-2164-9-461

**Published:** 2008-10-07

**Authors:** Jingkai Yu, Svetlana Pacifico, Guozhen Liu, Russell L Finley

**Affiliations:** 1 Center for Molecular Medicine and Genetics, Wayne State University School of Medicine, 540 East Canfield Ave., Detroit, MI 48201, USA; 2 Department of Biochemistry and Molecular Biology, Wayne State University School of Medicine, 540 East Canfield Ave., Detroit, MI 48201, USA

## Abstract

**Background:**

Charting the interactions among genes and among their protein products is essential for understanding biological systems. A flood of interaction data is emerging from high throughput technologies, computational approaches, and literature mining methods. Quick and efficient access to this data has become a critical issue for biologists. Several excellent multi-organism databases for gene and protein interactions are available, yet most of these have understandable difficulty maintaining comprehensive information for any one organism. No single database, for example, includes all available interactions, integrated gene expression data, and comprehensive and searchable gene information for the important model organism, *Drosophila melanogaster*.

**Description:**

DroID, the *Drosophila *Interactions Database, is a comprehensive interactions database designed specifically for *Drosophila*. DroID houses published physical protein interactions, genetic interactions, and computationally predicted interactions, including interologs based on data for other model organisms and humans. All interactions are annotated with original experimental data and source information. DroID can be searched and filtered based on interaction information or a comprehensive set of gene attributes from Flybase. DroID also contains gene expression and expression correlation data that can be searched and used to filter datasets, for example, to focus a study on sub-networks of co-expressed genes. To address the inherent noise in interaction data, DroID employs an updatable confidence scoring system that assigns a score to each physical interaction based on the likelihood that it represents a biologically significant link.

**Conclusion:**

DroID is the most comprehensive interactions database available for *Drosophila*. To facilitate downstream analyses, interactions are annotated with original experimental information, gene expression data, and confidence scores. All data in DroID are freely available and can be searched, explored, and downloaded through three different interfaces, including a text based web site, a Java applet with dynamic graphing capabilities (IM Browser), and a Cytoscape plug-in. DroID is available at .

## Background

Many of the important properties of biological systems emerge as a result of the interactions among genes and among their protein products. Genes and the proteins they encode participate in gene-gene, gene-protein, and protein-protein interactions to mediate a wide variety of biological processes. An increasing appreciation for the importance of charting these interactions has lead to many large-scale efforts to identify gene and protein interactions for a number of systems [1]. As this data continues to accumulate from a variety of sources there is an increasing need for comprehensive databases and analysis tools that allow biologists to make use of it. Genes and proteins that function in the same pathway, for example, interact directly or indirectly, and their functions can only be fully understood in the context of the interaction networks to which they belong.

Gene and protein interaction data have come from a variety of sources. To detect protein-protein interactions, for example, high throughput yeast two-hybrid [2-5] and co-affinity purification [6,7] screens have been developed and applied to proteins from humans and several model organisms. To generate large networks of gene-protein interactions, high throughput techniques are being developed for detecting transcription factors and other proteins bound to DNA [8-11]. Finally, gene-gene interactions that suggest functional relationships between pairs of genes are being revealed by large-scale assays for genetic interactions [12,13]. While each type of interaction data has proven useful for understanding how genes and their products work together in biological systems, the large amount of disparate data can be difficult to access and interpret. Combining data from different sources has become important because no single screen or technique is free from false positives and false negatives. Many studies have shown, for example, that interactions detected in multiple screens or by multiple techniques are less likely to be false positives (e.g., [14]), so that combining datasets can provide a simple way to gain confidence in any particular set of interactions. Likewise, the inability of any one technique or particular screen to detect all biologically relevant interactions suggests that combining datasets increases coverage.

A number of centralized databases have been implemented to store gene and protein interaction data and to make it publicly available [15-21]. While most of the data are from large-scale screens, several of these databases have also begun to include data from small-scale 'low throughput' experiments collected by manual curation of the literature. Despite the ideal of central databases to be comprehensive, a surprising number of interactions can be found in one database but not another [22,23]. Thus, biologists have been well advised to consult multiple databases to get a complete picture of the available data. Most of the large interaction databases include data for many different species. Such multi-species databases, however, are rarely fully comprehensive for any one organism; for example, organism-specific gene information, such as gene expression and phenotype data is not available for searching and filtering the interaction data. Multi-organism databases also have difficulty representing potentially conserved interactions for any given species. Finding conserved interactions requires looking up the orthologous proteins and conducting searches for interaction data in each of several different organisms. Recently, a few public databases have addressed these issues in efforts to generate comprehensive resources for a particular species; e.g., HomoMINT [24] and UniHI for humans [25]. DroID is designed to be a comprehensive interactions database dedicated to the important model organism, *Drosophila melanogaster*.

## Construction and content

### Database overview

**Figure 1:**
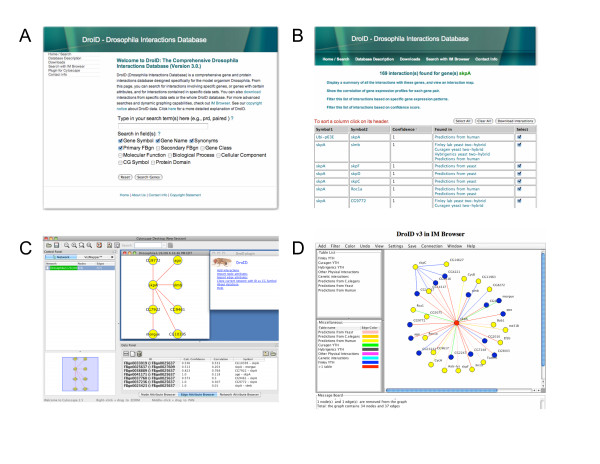
**DroID access interfaces**. (A) DroID search page; (B) Interaction search results page from which results can be filtered using gene expression data or confidence scores; (C) Cytoscape plug-in, which queries DroID directly and enables network visualization and analysis within Cytoscape; (D) IM Browser interface with dynamic graphing capabilities. All interfaces can be accessed from .

We developed DroID with several guiding principles in mind. First, we set out to combine all available gene and protein interaction data for *Drosophila *into one place where it could be frequently updated. DroID also contains searchable gene information from Flybase, the central repository for *Drosophila *gene information [26], enabling users to find or filter interactions based on *Drosophila*-specific gene attributes. Second, DroID strives to include all original data when available. For example, the database tries to obtain and store even technique-specific or experiment-specific details. These details, which are often missing from centralized databases, can facilitate a wider range of downstream analyses. Third, DroID tracks primary sources and secondary sources, providing links to references where available, so that users can trace the provenance of each interaction. Fourth, DroID strives to eliminate redundancy. If an interaction derived from a single primary reference is found in slightly different forms in multiple databases, a single instance with the appropriate reference appears in DroID. Fifth, DroID includes interactions predicted from experimental data for other major model organisms and humans. Because interactions are often conserved, data from other organisms can be used to infer likely interactions between orthologous proteins in *Drosophila*. Such predicted interactions, which have been called interologs [27], essentially enable researchers to use humans and other organisms as 'model organisms' for *Drosophila *studies. Sixth, every interaction in DroID is annotated with a confidence score providing a measure of the likelihood that it is a biologically relevant interaction, and a separate score indicating the level of co-expression of the two genes involved. Finally, we set out to provide complete access to DroID with three user-friendly interfaces that include some features especially geared toward *Drosophila *researchers (Figure 1).

DroID is an extensive update of an earlier database [28]. New features that are described in more detail below include a web interface, gene expression data, calculated gene correlation values, confidence scores, and substantially more interaction data. In addition, DroID is updated quarterly and each version is available for download. The current version of DroID (v4.0) is described here.

### Interaction data and generation of interologs

DroID is stored in a relational database with each major interaction dataset corresponding to one database table (see Table 1). As new datasets become available, new tables are added. The different datasets can be seamlessly integrated or searched separately. Frequently, the overlap among different datasets contains more reliable interactions, and this overlap will be obvious to users. While much of the data in DroID represents protein-protein interactions, all interactions are keyed to gene or locus identifiers because protein interaction data rarely includes knowledge of specific alternative splice forms or protein isoforms. DroID uses the Flybase gene number (FBgn) to specify a gene or a protein encoded by a gene. Other common gene identifiers, such as the gene symbol or CG number, are also stored.

**Table 1:** DroID interaction datasets

Data set	Number of Interactions	Number of Genes
Curagen yeast two-hybrid	20182	6875
Finley yeast two-hybrid	2915	1225
Hybrigenics yeast two-hybrid	1856	1282
Other physical interactions*	897	628
Human interologs	40548	3996
Yeast interologs	64407	2668
Worm interologs	2383	1432
Genetic interactions	5350	1644

* Physical interactions that were detected outside the three large-scale yeast two-hybrid screens. The two-hybrid datasets are from references [30] for Curagen, [29] for Finley, and [31] for Hybrigenics.

DroID contains the yeast two-hybrid interactions published in three major studies [29-31] in addition to unpublished interactions from an ongoing large-scale two-hybrid screening project [29,32]. Full experimental details as reported by the original publications are included. For *Drosophila *physical interactions not covered by the three large-scale yeast two-hybrid screens and for interactions of human, worm (*C. elegans*), and yeast proteins, raw data are downloaded from respective online databases. These databases include BioGRID [17], IntAct [16], and MINT [18], in addition to MIPS [33] for yeast and HPRD [20], PDZbase [34], and Reactome [21] for human. To enable periodic updates we established a pipeline for entering data into DroID as follows. First, raw interaction data is parsed to ensure that it includes only physical protein-protein interactions. DroID obtains interactions annotated with at least one detection method that detects physical interactions (e.g., yeast two-hybrid, mass spectrometry, pull down, etc.). Second, we map genes to uniform identifiers for the four organisms utilized by DroID; that is, Flybase gene number (FBgn) for fly, Ensembl gene identifier (ENSG) for human, Wormbase gene identifier (WBGene) for worm, and ORF identifiers for yeast. For each interaction, DroID stores the original PubMed identifier (PMID), methods used in detecting it, and the databases reporting it. Finally, we map interactions collected from human, worm, and yeast to *Drosophila *interologs by orthology mapping using Inparanoid (currently at version 6) [35]. DroID also stores genetic interactions obtained from Flybase, each annotated by reference numbers that trace to original data sources. Aside from interologs, DroID currently does not include interactions based solely on computational predictions, which may be found in other databases [36,37]. For example, the Fly-DPI database has *Drosophila *protein interactions predicted on the basis of domain pairs found in experimental PPI [37].

### Gene attributes and gene expression data

DroID includes a searchable gene attributes table populated from periodically updated gene annotations available in Flybase [26]. Users can search for interactions involving specific genes by searching for gene names, symbols, synonyms, or gene identifiers. The gene attributes table also allows searches based on gene class, gene function annotations based on gene ontology (GO) [38], and protein domains. The IM Browser interface [28] further extends this search ability by enabling a live search of Flybase for genes based on additional attributes, including reference and phenotype.

DroID also stores searchable gene expression data, which allows interaction data to be viewed and filtered in the context of gene expression patterns. DroID currently has two microarray-based gene expression datasets that can be used to constrain a search for interactions. One dataset includes genome-wide expression profiles over the course of embryogenesis in half-hour increments [39], and the other includes expression profiles for a developmental time course from early embryos through adults [40]. DroID can accommodate additional gene and protein expression data as they become available.

### Gene expression correlation

Genes that are frequently co-expressed often function together in common processes (e.g., [41,42]). Thus, there is substantial value in knowing the level of co-expression for pairs of genes that interact. To facilitate co-expression analyses for *Drosophila*, we computed correlation values between pair-wise expression profiles derived from the Gene Expression Omnibus (GEO) database [43]. We downloaded all *D. melanogaster *gene expression datasets from GEO and computed linear Pearson correlations between pair-wise expression profiles within each dataset. We first removed datasets that have less than 5 samples (e.g., tissues, conditions, or time points) to avoid possible spurious strong correlations. This resulted in 49 genome-wide expression datasets with 844 combined samples. Multiple correlations for a pair of genes from different datasets were then combined to produce a final correlation value for a specific gene pair. The combination is done based on how many samples each dataset has. Intuitively, a correlation based on a dataset having many samples may be more significant than the same value derived from another dataset with only a few samples. If there are *n *datasets, and each reports a correlation of x_i _for a gene pair, the final correlation value is computed by

corr = Σ_i_(x_i _* s_i_)/Σ_i_(s_i_)

where i ∈ [1, 2, ..., n], and s_i _represents the number of samples in data set i. Every interaction in DroID is annotated with the current gene expression correlation value for that pair. Correlation values are updateable as new gene expression datasets are added to GEO.

### Cross data set confidence scores

Protein-protein interaction data tend to be noisy, with variable rates of false positives from one dataset to another. A novel feature of DroID is the annotation of each physical protein-protein interaction with an updateable confidence score that reflects the probability that it is a biologically relevant true positive. Most methods for generating confidence scores work within a single type of data, such as yeast two-hybrid or protein complex data, by searching for features of the data that correlate with biological significance [30,44-47]. As a consequence, the scores derived for one data set bear little relation to those for another data set. In contrast, DroID assigns confidence scores to all physical interactions, including data from different techniques and interologs derived from worm, yeast, and human. The method used to assign confidence scores is based on the logistic regression approach described by Giot et al. [30,44]. In this approach we first identify training data, including a set of interactions that are likely to be true positives and another set that are likely to be false positives. We then search for specific attributes of the interactions that correlate with the two training sets. The attributes include gene expression correlation, number of associated literature citations (PubMed identifiers or PMIDs), local and global network topology, and domain-domain interactions. For example, the number of PMIDs for an interaction correlates with its likelihood of being in the true positive training set. Conversely, the number of interactions for a protein is inversely correlated with presence in the true positive training set. The correlations are then used to train a logistic regression model that can assign scores to all interactions based on their attributes. For the interactions in DroID, we used a variation of this scoring system in which we combine multiple training datasets to reduce the potential bias of any single training set (Yu, submitted).

Every physical interaction in DroID has a confidence score between 0 and 1 to represent the probability that it is a biological true positive. Validation of the scoring system shows that interactions with higher scores are more likely to be biologically relevant than interactions with lower scores (Yu et al., submitted; [44]). The set of interactions with scores greater than 0.5, for example, have significantly more pairs of genes that share GO biological process or cellular component annotations compared to interactions scoring < 0.5, or to random pairs. Interactions scoring less than 0.5 also share significantly more GO annotations than random pairs of genes, which indicates that overall the interactions collected in DroID are enriched for biologically relevant true positives. As additional interaction data and other new information become available, the scoring models can be periodically retrained to improve the overall accuracy. Thus, the confidence scores are updateable and receive a version number at each revision.

## Utility and discussion

### Summary of the database

The current version of DroID (v4.0) contains 131,659 links among 9,511 *D. melanogaster *genes, or roughly 64.4% of the predicted genes. The small amount of overlap between different interaction sets (Table 2) shows that no single dataset adequately covers the available data, and serves to illustrate the value of making all data available in one location. A major limitation to the value of most interaction datasets is the presence of false positive interactions that have no biological significance. To overcome this limitation and to help biologists focus on the most reliable interactions, DroID assigns confidence scores to individual interactions to denote potential biological significance. The current version of the confidence scoring system (v2.0) assigned scores to the 126,896 physical interactions in DroID (excluding genetic interactions). Of these, 28,259 (22.3%) interactions received a score above 0.5, distinguishing them as the high confidence set. These scores should help biologist focus on the most reliable subset of the data for future studies. For example, networks and subnetworks can be filtered based on user-defined confidence limits to accommodate analyses that tolerate different levels of uncertainty.

**Table 2:** Overlap between interaction datasets

	Worm	Yeast	Human	Genetic	Other*	Hybrigenics	Curagen
Finley	14	73	118	15	6	4	48
Curagen	69	168	263	35	48	23	
Hybrigenics	8	15	54	24	9		
Other*	12	52	220	182			
Genetic	8	55	403				
Human	201	4830					
Yeast	137						

* Physical interactions that were detected outside the three large-scale yeast two-hybrid screens.

### Gene expression correlation

**Figure 2:**
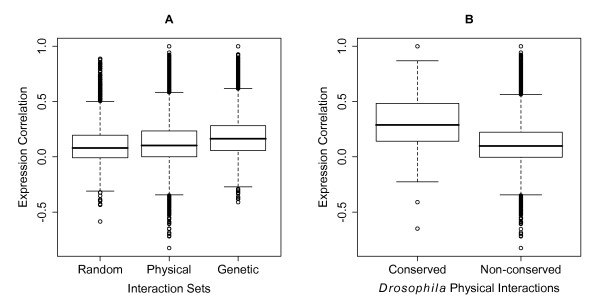
**Expression correlation comparison**. Boxplots of average expression correlations of different interaction sets. (A) Comparison between random pairs of genes not known to interact (Random), all physical interactions in DroID (Physical), including interactions detected with *Drosophila *proteins and those predicted based on human, worm, and yeast physical interactions (Table 1), and genetic interactions (Genetic). (B) Difference of expression correlations between putative conserved and putative non-conserved fly physical interactions. The conserved set includes *Drosophila *physical interactions that overlap with any of the three sets of predicted physical interactions, while the non-conserved set contains the rest of the physical interactions.

**Figure 3:**
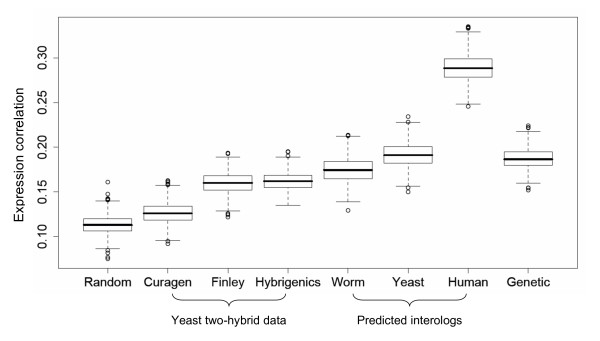
**Gene expression correlation for interaction data sets in DroID**. Boxplots of correlation distributions of different data sets show that all sets of interactions in DroID have higher average expression correlation than the set of random protein pairs. The X-axis represents various sets of interactions in DroID, 'Random' denotes sets of random protein pairs. The Y-axis represents expression correlations.

In addition to physical protein-protein interactions and genetic interactions, gene expression data can be a valuable tool for linking together genes that may function together. It has been shown, for example, that genes with correlated expression patterns are more likely to function together in common biological processes (e.g., [41,42]), and at least in yeast, proteins encoded by co-expressed genes are more likely to participate in direct physical interactions than random pairs [48]. To help reveal relevant functional linkages, every gene pair in DroID is annotated with gene expression correlation values. Consistent with findings in yeast, we found that the physical protein-protein interactions in DroID are encoded by gene pairs with significantly higher expression correlations than random gene pairs (p-value < 2.2*10^-16^, Figure 2A and Figure 3). In addition, higher expression correlation values were seen for gene pairs that genetically interact, and therefore are likely to function in common biological processes (Figure 2A). Interestingly, the *Drosophila *physical interactions that overlap with interologs detected in other species have a significantly higher expression correlation than the remainder of the physical interactions (p-value < 2.2*10^-16^, Figure 2B), suggesting that conserved interactions involve proteins that are more likely to be co-expressed than non-conserved interactions. It is noteworthy that the average correlation values are not very high (e.g., 0.13 for the DroID physical interactions) and that many gene pairs have a negative correlation. This result is not surprising for a multi-cellular organism in which functionally relevant interactions can occur between pairs of proteins even if they are only co-expressed during a fraction of developmental time or in just one or a few tissues.

### Viewing interaction data in the context of gene expression data

Gene expression data can also be used to view interaction data in a dynamic context. Most gene and protein interaction data that are currently available come from studies that are independent of gene expression. Examples include yeast two-hybrid data in which pairs of proteins are expressed together in yeast, whether or not they are co-expressed *in vivo*, and co-AP experiments in which often at least one of the proteins is artificially expressed with an affinity tag in tissue culture cells. Thus, the protein interactions in DroID and most other databases represent pairs of proteins that *may *interact *in vivo*, but only if they are expressed together. A powerful way to view this interaction data, therefore, is in the context of gene expression patterns for a particular tissue or developmental time point. DroID includes gene expression data from genome-wide developmental studies. This data can be used to constrain a set of interactions to include only genes expressed at a user-defined level and time point or developmental stage.

### DroID access interfaces

All data in DroID can be accessed and downloaded in part or whole via three different interfaces (Figure 1). A user-friendly web interface is provided for simple searching, browsing, and downloading of DroID data. Going to the DroID web page opens a search box, which asks users for a term describing a gene or protein. The term can be a gene symbol, name, synonym, or a term describing a gene or protein (Figure 1A). Clicking 'Search Genes' produces a page listing genes that fit the search criteria. On this page, users select one or any number of the genes, and then have the option to select specific interaction datasets or to search all of them simultaneously. The search produces a results page listing the found interactions and their current confidence scores (Figure 1B). Each interaction is represented by the symbols of the two genes and a list of the datasets in which they were found. Additional information about each gene, including GO annotations and links to Flybase can be obtained by clicking on the gene symbol. Similarly, clicking on the dataset name for each interaction reveals its details, including original experimental data when available, references, and relevant links. The results page also includes several additional options for further analysis. These include an option to show the gene expression correlation values for each interaction and an option to filter the results by gene expression patterns or confidence scores. Utilizing these filters helps researchers to focus on interactions that are more likely to be true positives or that involve co-expressed genes. The results page also includes a link for downloading the interactions in formats that can then be uploaded into network analysis programs. Finally, a link is included that will generate a summary table showing the number of interactions for the selected genes in each of the interaction datasets, including those not originally searched. The summary table also includes a button that automatically opens the IM Browser applet to generate a graphical map of the interactions (see below).

DroID can be accessed via two different dynamic interfaces that allow an interaction network to be explored as a graph where nodes represent genes or proteins and edges connecting the nodes represent interactions. Viewing an interaction map in this way places each gene and interaction into the context of other interactions and facilitates biological insights that are not possible from simple lists of interactions. The first interface is a plug-in (Figure 1C) that allows DroID to be accessed through the powerful network visualization and analysis program, Cytoscape [49]. The second interface is IM Browser (Figure 1D), a program originally designed to access an earlier version of DroID and other interaction databases [28]. IM Browser runs as a java applet and allows advanced queries and dynamic graphing of search results. While a complete description of IM Browser capabilities is beyond the scope of this paper, a few features are worth noting here. First, the program easily accommodates new types of interaction data and dynamically enables all node and edge information to be used in searches and filtering. This feature is important as new techniques for detecting interactions are needed and continue to emerge, and each new technique has its own type of data. Second, interaction maps can be edited and saved to the user's local computer, and local datasets can be loaded into the program to allow the user to view and analyze their own interactions in the context of DroID data. Finally, a new feature of IM Browser allows maps to be filtered based on gene expression data or confidence scores. The constraint is implemented as a dynamic filter that can be applied to an existing interaction map. As new gene expression data becomes available, and eventually protein expression data is collected from proteomics studies, an increasingly fruitful way to view interaction maps will be in the context of specific temporal and spatial expression patterns.

## Conclusion

DroID is a comprehensive interactions database designed specifically for *Drosophila melanogaster*. The database currently covers more *Drosophila *genes and interactions than any other single database and is periodically updated. Because it is an organism-specific database, it readily includes potentially conserved interactions found in other organisms by mapping them to *Drosophila *genes. The database also includes comprehensive gene information, including *Drosophila*-specific information, which can be used to search for and filter interactions and to analyze gene networks. DroID includes gene expression data, both as expression profiles and as correlation values, to help researchers link together genes that may function together in specific biological processes. Finally, DroID assigns updateable confidence scores to every physical interaction to help focus studies on biologically relevant links. Combined with three user interfaces, DroID should provide a valuable resource for studying *Drosophila *systems.

## Availability and requirements

DroID is freely available for non-commercial use. Any modern web browser can access the DroID home page at . A web browser with an installed Java Virtual Machine can access IM Browser from the DroID home page or from a list of found interactions. Cytoscape [49] enables installation and usage of the DroID plugin for Cytoscape.

## Authors' contributions

RLF conceptualized the project. JY and RLF designed and built the database. JY performed data analysis. SP and RLF designed and built the interfaces. GL compiled a previous version of the database. RLF and JY wrote the draft. All authors tested the database and interfaces.
